# French Scorpionism (Mainland and Oversea Territories): Narrative Review of Scorpion Species, Scorpion Venom, and Envenoming Management

**DOI:** 10.3390/toxins14100719

**Published:** 2022-10-21

**Authors:** Jules-Antoine Vaucel, Sébastien Larréché, Camille Paradis, Arnaud Courtois, Jean-Marc Pujo, Narcisse Elenga, Dabor Résière, Weniko Caré, Luc de Haro, Jean-Christophe Gallart, Romain Torrents, Corinne Schmitt, Johan Chevalier, Magali Labadie, Hatem Kallel, French PCC Research Group

**Affiliations:** 1 Bordeaux Poison Control Centre, Centre Hospitalier et Universitaire Bordeaux Pellegrin, 33000 Bordeaux, France; 2 Medical Biology Department, Hôpital d’Instruction Des Armées Bégin, 94160 Saint-Mandé, France; 3 Institut National de la Santé et de la Recherche Médicale (INSERM) UMR-S 1144, Université de Paris, 75000 Paris, France; 4 Emergency Department, Centre Hospitalier de Cayenne, 97300 Cayenne, France; 5 Pediatric Unit, Centre Hospitalier de Cayenne, 97300 Cayenne, France; 6 Intensive Care Unit, Centre Hospitalier et Universitaire Martinique, 97200 Fort de France, France; 7 Paris Poison Control Center, Fédération de Toxicologie (FeTox), Hôpital Fernand Widal, AP-HP, 75000 Paris, France; 8 Internal Medicine Department, Hôpital d’Instruction des Armées Bégin, 94160 Val-de-Marne, France; 9 Marseille Poison Control Centre, Assistance Public des Hôpitaux de Marseille, 13000 Marseille, France; 10 Toulouse Poison Control Centre, Centre Hospitalier et Universitaire de Toulouse, 31000 Toulouse, France; 11 Microentreprise WANO Guyane, 97319 Awala Yalimapo, France; 12 Intensive Care Unit, Centre Hospitalier de Cayenne, 97300 Cayenne, France

**Keywords:** scorpion stings, scorpion venom, public health, poison control centers, intensive care units

## Abstract

Sixty-seven scorpion species have been described in France and its territories, where they have been found to be heterogeneously distributed. Indeed, only one species can be found on Réunion Island, while 38 species exist in French Guiana. The number of stings is also heterogenous, with up to 90 stings per 100,000 inhabitants occurring annually. Scorpion species can frequently be determined through simple visual factors, including species of medical importance (i.e., *Buthus, Centruroides* and *Tityus*). Scorpion venom is composed of local enzymes and peptides with a cysteine-stabilized α/β motif (NaTxs, Ktxs, Calcines), which allow for venom diffusion and the prey’s incapacitation, respectively. Harmful scorpion species are limited to *Centruroides pococki* in the French West Indies, which can induce severe envenoming, and the *Tityus obscurus* and *Tityus silvestris* in French Guiana, which can cause fatalities in children and can induce severe envenoming, respectively. Envenomation by one of these scorpions requires hospital monitoring as long as systemic symptoms persist. Typical management includes the use of a lidocaine patch, pain killers, and local antiseptic. In the case of heart failure, the use of dobutamine can improve survival, and pregnant women must consult an obstetrician because of the elevated risk of preterm birth or stillbirth. France does not have scorpion antivenom, as scorpion stings are generally not fatal.

## 1. Epidemiology

Scorpionic envenoming is common in tropical and subtropical areas, with an annual incidence estimated at 1,500,000 cases worldwide, which have led to 2600 deaths [1]. The number of deaths in North Africa from scorpion envenoming is the most concerning [2,3]. Indeed, 62% of scorpion sting deaths occur in North Africa [1], and they are mostly related to envenoming by the *Androctonus* genus scorpion *(Androctonus australis, Androctonus crassicauda,* and *Androctonus mauretanicus*) [4,5,6,7], *Hottentotta gentili* [7], and possibly the *Buthus* genus scorpion, but the species involved are actually not well identified [5,7,8]. Among all scorpions, only species from the family *Buthidae* are considered deadly to humans, with some rare exceptions [5]. There are several French departments and territories, including those in South America (French Guiana), the French West Indies (Guadeloupe, Martinique, Saint Barthélemy, and Saint Martin), the Indian Ocean (Réunion Island and Mayotte), the Pacific Ocean (New Caledonia, French Polynesia, Wallis, and Futuna Islands), the North Atlantic (St. Pierre and Miquelon), Antarctica (Terre Adélie), and Austral Ocean (Éparses Islands, Kerguelen Islands, Crozet archipelago, Saint Paul, and Amsterdam Islands). Within the entire territory, the frequency of scorpionic envenoming ranges from 0 to 90 stings per 100,000 inhabitants annually [9,10,11]. Envenoming by scorpion is described in all regions of continental France, except Brittany [9]. Higher sting frequencies occur in Provence-Alpes-Côte d’Azur, Occitania, and French Guiana. Of the cases of scorpion stings reported to the French poison control center, 10% are related to a non-native scorpion species. Some of these cases concern foreign countries, while others involve scorpions imported in an unintentional way (i.e., in luggage when returning from a trip or in international parcels) [9], or by imported scorpions bred as pets [12,13].

Among the 2200 species of scorpions referenced in the world, 67 are found in French territories (Table 1), with thirty-eight scorpion species in French Guiana, thirteen in the French West Indies, eight in the Pacific Ocean islands, eight in continental France, three in Corsica, and three in the Indian Ocean islands [5,9,14,15,16,17,18,19,20,21,22,23,24,25,26,27,28,29,30,31,32,33,34,35,36,37,38,39,40,41,42,43]. Adults are more often victims of scorpion stings, although children are most at risk of complications. Severe envenoming can occur in French Guiana and the French West Indies. In French Guiana, envenomation by *Tityus obscurus* in children under 12 years old can result in severe complications and even death [5,10,11,44,45,46,47]. In this population, 30% of envenomed patients show signs of severe complications, a proportion that increases to 60% in children under three years of age [45]. Indeed, cardiac symptoms were recorded in 82% of cases with general signs of envenomation. The presence of pulmonary; ear, nose, and throat (ENT); or gastrointestinal symptoms are related to major envenomation. The rare cases of death are related to acute left heart failure [45]. In French Guiana, *Tityus silvestris* can also induce severe envenoming [14,15].

In the French West Indies, envenomation by *Centruroides pococki* comes with a risk of severe complications, although no deaths have been reported to date [9,14,48,49]. In adults, envenomation can lead to ascending muscular paralysis, radiating from the point of sting and with progressive healing to the sting area [14].

In other territories, scorpionic envenomation causes only minor symptoms, mostly asthenia, hyperthermia, palpitation, tachycardia, paresthesia, nausea, and muscle weakness [9]. This is what has been observed in cases of envenomation by Euscorpiidae family scorpions or *Buthus* genus scorpions. Interestingly, the *Buthus occitanus, Buthus pyrenaeus,* or *Buthus balmensis* species are present only in Western Europe, [9,16,17,35,48,50,51,52,53], while the other species of *Buthus* from North Africa (i.e., *Buthus lienhardi*, *Buthus malhommei*, *Buthus mardochei*, *Buthus paris*, and *Buthus tunetanus*) can induce fatal envenomation [6,8,54,55]. The difference in sting severity between the different *Buthus* species is not understood, as their venom is similar [56]. Scorpionic envenomation remains understudied in France since it is considered harmless. However, severe complications can occur, and this review aimed to provide an overview of envenomation in metropolitan and overseas France, as well as a description of its management.

**Table 1:** Scorpion species present in France, envenoming classes (based on the consensus classification of the expert group on scorpions [57]) and their territorial distribution.

Family or Species	Envenoming Class	Continental France	Corsica	Guadeloupe	French Guiana	French Polynesia	Martinique	Mayotte	New Caledonia	Reunion Island	Reference
Belisariidae											
*Belisarius xambeui* (Simon, 1879)	I										[16,18]
Buthidae											
*Ananteris coineaui* (Lourenço, 1982)	- *										[19]
*Ananteris dacostai* (Ythier, Chevalier & Lourenço, 2020)	-										[20]
*Ananteris elisabethae* (Lourenço, 2003)	-										[19]
*Ananteris guyanensis* (Lourenço & Monod, 1999)	-										[19]
*Ananteris intermedia* (Lourenço, 2012)	-										[19]
*Ananteris kalina* (Ythier, 2018)	-										[19]
*Ananteris mamilihpan* (Ythier, Chevalier & Lourenço, 2020)	-										[20]
*Ananteris polleti* (Lourenço, 2016)	-										[19]
*Ananteris sabineae* (Lourenço, 2001)	-										[19]
*Ananteris sipilili* (Ythier, Chevalier & Lourenço, 2020)	-										[20]
*Ananteris tresor* (Ythier, Chevalier & Lourenço, 2020)	-										[20]
*Buthus occitanus* (Amoreux, 1789)	II										[5,16,18]
*Buthus balmensis* (Ythier & Laborieux, 2022)	-										[53]
*Buthus pyrenaeus* (Ythier, 2021)	I										[43]
*Centruroides barbudensis* (Pocock, 1898) **	I										[21,22,23]
*Centruroides gracilis* (Latreille, 1804)	-										[23]
*Centruroides pococki* (Sissom & Francke, 1983)	III										[14,21,23]
*Grosphus goudoti* (Lourenço & Goodman, 2006)	-										[24]
*Grosphus mayottensis* (Lourenço & Goodman, 2009)	-										[24]
*Isometrus maculatus* (De Geer, 1778)	II										[5,9,17,19,21,25]
*Jaguajir pintoi kourounensis* (Lourenço, 2008)	-										[19]
*Microananteris abounami* (Lourebço & Chevalier 2022)											[58]
*Microananteris inselberg* (Lourenço, 2021)	-										[42]
*Microananteris minor* (Lourenço, 2003)	-										[42]
*Microananteris serrulata* (Lourenço, 2021)	-										[42]
*Reddyanu heimi* (Vachon, 1976)	-										[39]
*Tityus gasci* (Lourenço, 1981)	-										[19]
*Tityus mana* (Lourenço, 2012)	-										[19]
*Tityus marechali* (Lourenço, 2013)	-										[26]
*Tityus metuendus* (Pocock, 1897)	-										[27]
*Tityus obscurus* (Gervais, 1843)	III										[5,9,19]
*Tityus silvestris* (Pocock, 1897)	III										[15,19]
Bothriuridae											
*Cercophonius squama* (Gervais, 1843)	-										[41]
Chactidae											
*Auyantepuia aluku* (Ythier, 2018)	-										[19]
*Auyantepuia aurum* (Ythier, 2018)	-										[19]
*Auyantepuia fravalae* (Lourenço, 1983)	-										[19]
*Auyantepuia gaillardi* (Lourenço, 1983)	-										[19]
*Auyantepuia kelleri* (Lourenço, 1997)	-										[19]
*Auyantepuia laurae* (Ythier, 2015)	-										[19]
*Auyantepuia sissomi* (Lourenço, 1983)	-										[19]
*Broteochactas delicatus* (Karsch, 1879)	I										[19,28]
*Brotheas gervaisii* (Pocock, 1893)	I										[19,28]
*Brotheas granulatus* (Simon, 1877)	I										[19,28]
*Guyanochactas flavus* (Lourenço & Ythier, 2011)	-										[19]
*Guyanochactas gonzalezspongai* (Lourenço, 1983)	-										[19]
*Guyanochactas touroulti* (Lourenço, 2018)	-										[29]
*Hadrurochactas cristinae* (Ythier, 2018)	-										[19]
*Hadrurochactas schaumii* (Kirsch, 1880)	-										[19]
*Spinochactas mitaraka* (Lourenço, 2016)	-										[19]
Diplocentridae											
*Didymocentrus martinicae* (Teruel & Questel, 2020)	-										[23,36]
*Oiclus ardens* (Ythier, 2019)	-										[31]
*Oiclus cousteaui* (Ythier, 2019)	-										[31]
*Oiclus nanus* (Teruel & Chazal, 2010)	-										[32]
*Oiclus purvesii purvesii* (Becker, 1880)	-										[31]
*Oiclus purvesii sabae* (Francke, 1978)	-										[31]
*Oiclus questeli* (Teruel, 2008)	-										[31]
*Oiclus tipunch* (Ythier, 2019)	-										[31]
Euscorpiidae											
*Euscorpius concinnus* (Koch, 1837)	II										[16,33]
*Euscorpius italicus* (Herbst, 1800)	II										[9,18,34,35]
*Euscorpius tergestinus* (Koch, 1837)	II										[9,18,34,35]
*Tetratrichobothrius flavicaudis* (De Geer, 1778)	II										[9,16,18,34,35]
Hormuridae											
*Hormurus neocaledonicus* (Simon, 1877)	-										[40]
*Opisthacanthus heurtaultae* (Lourenço, 1980)	-										[19]
Liochelidae											
*Liocheles australasiae* (Fabricius, 1775)	-										[25,40]
*Liocheles neocaledonicus* (Simon, 1877)	-										[38]
*Liocheles longimanus* (Werner, 1939)	-										[40]
Total: 67 species		8	3	11	39	2	6	3	7	1	

A black square corresponds to the presence of the scorpion species on the territory described above. * The notation “-” corresponds to an absence of information about envenoming class; ** the presence of Centruroides barbudensis (Pocock, 1898) in Martinique is uncertain [23].

## 2. Visual Determination of Scorpion Species for Medical Management

**Figure 1:**
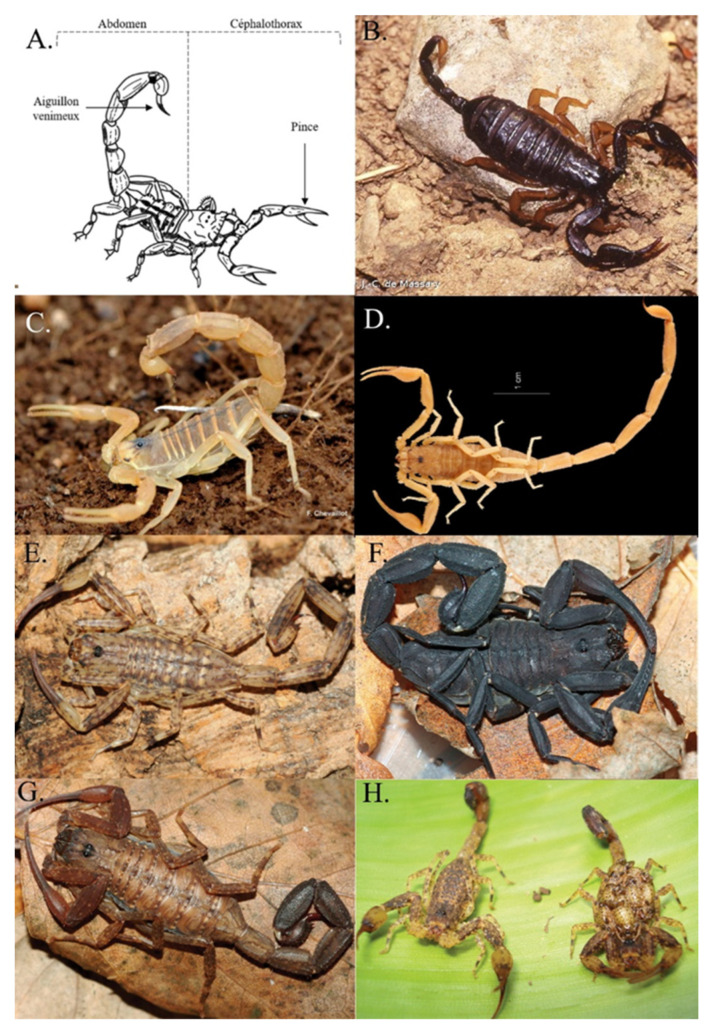
Brief anatomical description of a scorpion and photos of scorpions of medical interest present on French territory: (**A**) anatomical description of a scorpion; (**B**) *Tetratrichobothrius flavicaudis;* (**C**) *Buthus occitanus*.; (**D**) *Centruroides pococki*; (**E**) *Isometrus maculatus*; (**F**) *Tityus obscurus* adult; (**G**) *Tityus obscurus* juvenile; (**H**) *Tityus silvestris*. (**B**–**D**) are available on the site https://inpn.mnhn.fr (accessed on 1 October 2022.) under Creative Commons license: (**B**) by J.-C. De Massary, (**C**) by F. Chevaillot, and (**D**) by E.-A. Leguin. (**E**–**H**) are already published [19] and they are presented here with the permission of E. Ythier.

The identification of the species involved in a scorpion sting is helpful for optimal medical management. Following a scorpion sting, it is often possible to determine the species, or at least the genus, through visual descriptions and the location of the incident. Important considerations include the size, color, and appearance of the scorpion’s pincers (i.e., thin or thick; Figure 1A).

### 2.1. Metropolitan France (Continental France and Corsica)

Eight species of scorpions have been recorded in metropolitan France: three *Buthus*, one *Tetratrichobothrius*, three *Euscorpius*, and one *Belisarius* [16,59,60]. Scorpions of the family *Euscorpiidae* (Figure 1B) are identifiable by color, with the body, claws, and tail being black and the legs a translucent yellow. Species belonging to the family *Euscorpiidae* can only be identified by an entomologist who analyzes the presence of ventral silky hairs on the claws, called trichobothria [61]. The genus *Buthus* (Figure 1C) is easily recognizable by its yellow color and a body that can turn gray. The presence of dark central longitudinal spots (called tergites) on the back of *Buthus pyrenaeus* allows it to be distinguished from *Buthus occitanus*. *Buthus pyrenaeus* is found above an altitude of 500 m, unlike *Buthus occitanus*. *Belisarius xambeui* is an orange–brown scorpion living only in the heights of the Pyrénées-Atlantiques and is rarely responsible for envenoming [9,52].

### 2.2. The French West Indies

Thirteen species of scorpions have been recorded in the French West Indies: three *Centruroides*, one *Didymocentus*, one *Isometrus*, seven *Oiclus*, and one *Tityus*. The size of the pincers can be used to differentiate the genus *Oiclus* (large pincers) from the genus *Centruroides*, which has fine pincers, as noted with *Centruroides pococki* (Figure 1D). In Guadeloupe, it is easy to differentiate *Isometrus maculatus* (Figure 1E) due to its spotted body. In Martinique, *Didymocentus martinicae* can be identified by its plain, dark-brown color and large claws. *Isometrus maculatus* and *Tityus marechali* are difficult to differentiate without examination by a specialist. Recent studies have cast doubt on the presence of the genus *Centruroides* in Martinique [23].

### 2.3. The islands of the Indian and Pacific Oceans

In the Indian and Pacific Ocean islands, ten species of scorpions are referenced: one *Cercophonius*, two *Grosphus*, two *Hormurus*, three *Liocheles*, one *Isometrus*, and one *Reddyanu*. Réunion Island hosts only one species, the *Isometrus maculatus*. In Mayotte, *Isometrus maculatus* is distinguished from the genus *Grosphus* by its spotted body. However, the similarity in color and distribution between the species of *Grosphus* does not allow for the determination of the species involved in envenomation [24]. In French Polynesia, the visual difference is easily observed between *Liocheles australasiae* (uniform color) and *Isometrus maculatus* (spotted). *Isometrus maculatus* is easily distinguished by its spotted body in New Caledonia.

### 2.4. French Guiana

Thirty-eight species of scorpions are found in French Guiana and include eleven *Ananteris*, seven *Auyantepuia*, one *Broteochactas*, three *Brotheas*, three *Guyanochactas*, two *Hadrurochactas*, one *Isometrus*, one *Jaguajir*, three *Microananterus*, one *Opisthacantus*, one *Spinochactas*, and five *Tityus*. The visual similarity and distribution of the 38 scorpion species do not allow for the determination of the species without capture, let alone the identification of the genus of the scorpion, except for the adult *Tityus obscurus* [19]. Indeed, only adult *Tityus obscurus* can be distinguished by their black color, large size, and fine pincers (Figure 1F). In all other situations, black pigmentation or the presence of fine pincers, analyzed independently, do not meet the criteria for the identification of a dangerous scorpion species. Indeed, “fine pincers” are difficult to identify with an untrained eye and not all envenomation by *Tityus obscurus* is easily identifiable. Indeed, juvenile *Tityus obscurus* are brown and can be confused with other species (Figure 1G). The other dangerous scorpion species, *Tityus silvestris* (Figure 1H), is yellow with brown spots and can be confused with other scorpion species. Therefore, it is difficult to rely on a visual description to determine the species or genus of scorpions in French Guiana.

## 3. Scorpion Venom

### 3.1. Generality

The venom of scorpions is synthesized by specialized glands in the telson, which is located in the terminal part of the tail. The telson morphology is quite similar between scorpion species, with rare exceptions. It is composed of a vesicle that contains a pair of glands; this vesicle is prolonged by the aculeus, which bears two exit ducts, each corresponding to one of the glands [62]. Venom is injected through the sting by the contraction of a striated skeletal muscle present in the venom gland. In 2019, there were approximately 550 scorpion toxins described [63].

Firstly, local enzymes act on tissue and vascular permeability to allow venom diffusion, which are also responsible for local pain, but they do not cause necrotic lesions. The enzymes included are hyaluronidases, phospholipases, and metalloproteases [64,65,66]. Hyaluronidases act by disrupting the integrity of the extracellular matrix and connective tissues [67]. Phospholipases hydrolyze phospholipids, which result in the disruption of cell membranes [65]. Finally, metalloproteases act mainly as hemorrhagic factors, also called hemorrhagins, which exert their effects by degrading proteins such as laminin, nidogen, fibronectin, collagen type IV, and proteoglycans from the endothelial basal membrane [68]. There are also adenosine, leucyl-tryptophan, and isoleucyl-tryptophan, which are platelet antiaggregants that increase the action of enzymes and are responsible for rare cases of local hematoma [65]. Modifications to tissue permeability also favor the superinfection of soft body parts, which is not often severe and may resolve spontaneously. Rare cases of skin infections appear in the week following the envenomation. If a skin infection is diagnosed, antibiotic therapy with beta lactams allows for the effective resolution of the infection. These complications are described for all scorpion species within the entire French territory [9].

Secondarily, peptides with a cysteine-stabilized α/β motif and three of four disulfide bridges, known as Knottins, are at the origin of the other symptoms described below. These are large toxins that target sodium channels (NaTxs), as well as small toxins that target potassium channels (KTxs) and calcium-release channel-specific peptides (calcines) [69].

**Figure 2:**
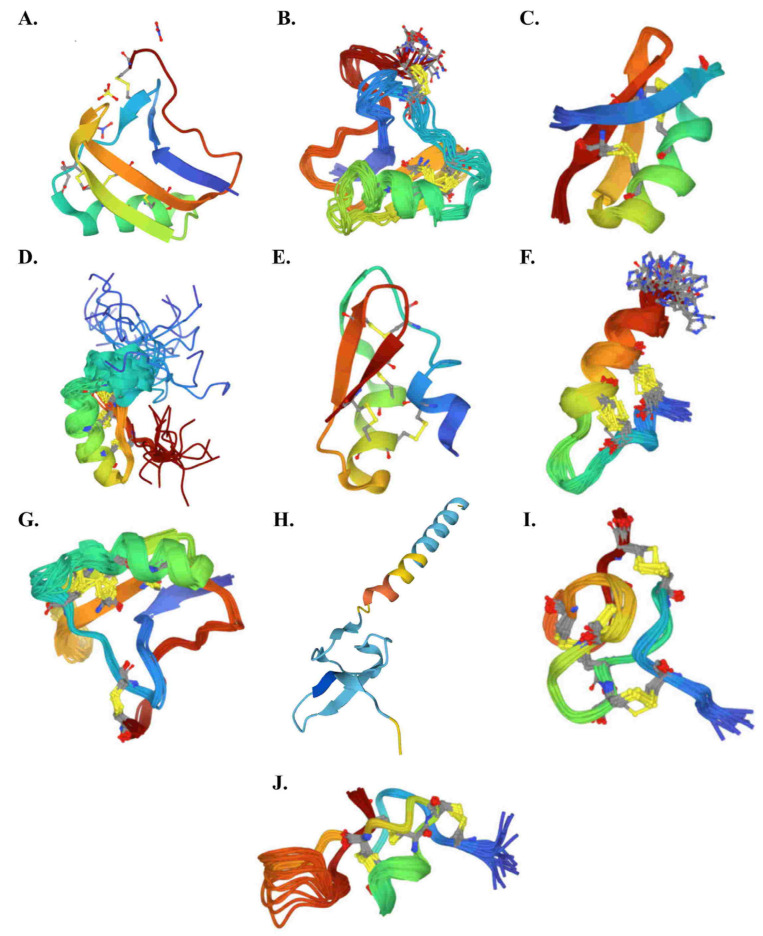
3D structure diagrams of typical cysteine-stabilized α/β motif peptides of scorpion venom obtained from https://www.rcsb.org/ (accessed on 1 October 2022): (**A**): classical α-NaTx (AaH II from *Androctonus australis hector*); (**B**): classical β-NaTx (Cn2 from *Centruroides noxius*); (**C**): classical α-KTx toxin (Agitoxin 2 from *Leiurus quinquestriatus hebraeus*); (**D**): classical β-KTx toxin (β-KTx14.3 from *Lychas mucronatus*); (**E**): classical γ-KTx (CnErg1 Ergtoxin from *Centruroides noxius*); (**F**): classical κ-KTx toxin (HelaTx1 from *Heterometrus laoticus*); (**G**): classical δ-KTx (BmK-M10 from *Mesobuthus martensii*); (**H**): classical λ-KTx (λ-MeuTx from *Mesobuthus eupeus*); (**I**): classical ε-KTx (TsPep1from *Tityus serrulatus*); and (**J**): classical calcine (Agitoxin A from *Pandinus imperator*).

NaTxs are 55 to 76 amino acid peptides (6500 to 8500 Da) with generally three of four disulfide bridges according their special arrangement [70]. They are divided in α and β-NaTxs according the interaction site on 3 or 4 sodium ion channels, respectively (Figure 2A,B) [65,71]. Another classification proposes 14 NaTxs subfamilies named NaTx1 to NaTx14 based on the alignment of cysteine residues and other highly conserved domains [72]. They are mostly described in the scorpion venom of the old world and new world, respectively. α and β-NaTxs are subdivided in three (antimammals, anti-insect, and active on both insect and mammals) and four (antimammals, anti-insect and mammals, anti-insect selective excitatory, and anti-insect selective depressant) groups, respectively [73]. In *Buthidae* family scorpions, 4000–8000 putative NaTxs should exist [70]. Therefore, they could not be detailed here.

KTxs are 23 to 64 amino acid peptides (less than 4000 Da), and thermostable [65,74,75] KTxs can be divided into α, β, γ, κ, δ, λ, and ε-KTx groups (Figure 2C–I) [76,77]. They specifically bind to the pore loop in potassium channels, especially the Kv1.1, Kv1.2, Kv1.3, and hERG channel [63]. Each of them presented some specificity. The α-KTx group, with 23–42 amino acid peptides, is the largest subgroup, and act as potassium ion channel blockers. The β-KTx group, with 50–57 amino acid peptides, act as potassium ion channel blockers. γ-KTx mainly blocks the ether-à-go-go-Related Gene (hERG) channel. δ-KTxs exerts both protease and potassium channel-inhibiting properties. The λ-KTx group contains an inhibitor cystine knot. Finally, ε-KTx, which was recently described, contains an inhibitor cystine knot motif [65]. A manually curated Ktx database was available at http://kaliumdb.org/ (accessed on 1 October 2022) [78].

Cacines are 33 amino acid peptides (3.7 to 4.2 kD) and are highly basic (pI 8.9 to 10.1), globular, highly soluble in water (hydrophilicity = 0.7–1.2), and thermostable (Figure 2J). The eight calcines (i.e., hadrucalcin, hemicalcin, imperacalcin, maurocalcin, opicalcin_1_, opicalcin_2_, urocalcin, vejocalcin) show high similarity (≥78.8% identity), which include the presence of three disulfide bridges along with sections of polypeptide between them forming β strands. They activate the Ca^2+^ release channel/ryanodine receptors (RyRs, intracellular ligand-activated calcium channels found in sarcoplasmic reticulum membranes) by stimulating [^3^H]ryanodine binding with a high affinity (*K*_d_ ≈ 5–10 nM) and specificity, which leads to an increase in the intracellular Ca^2+^ level and subsequently contractile paralysis [65,79].

### 3.2. French Scorpion Venom

The venom of French scorpions is poorly studied. This is due to many reasons, the main one being the low lethality of scorpion envenomation. As in the rest of the world, the venom studies are focus on the *Buthidae* family scorpion.

**Figure 3:**
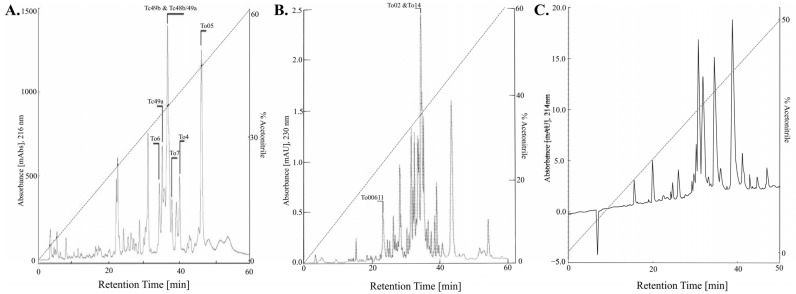
High performance liquid chromatography separation soluble venom. (**A**): *Tityus. Obscurus* venom from Guerrero-Vargas and et al [72]; (**B**): *Tityus metuendus* venom from Batista and et al [87]; (**C**): *Buthus occitanus* venom from Martin-Eauclaire and et al [56].

*Tityus* genus scorpions are the most harmful scorpions in France. However, *Tityus obscurus* and *Tityus silvestris* are neither as threatening nor as frequent a source of stings, nor as studied, as *Tityus serrulatus*, *Tityus bahiensis*, and *Tityus stigmurus* present in South America [80]. The venom of *Tityus obscurus, formerly named Tityus cambridgei, was first studied in* 1994 and had the highest lethal dose in mice among the Brazilian *Tityus scorpions* studied, with a 50 % lethal dose (LD50) at 12.136 mg/kg [81]. In 2000, more than 60 components were individualized and Tc1 (*Tityus cambridgei* toxin 1 o α-KTx13.1) was first described (Figure 3A). The *Tityus obscurus* venom was similar between all the specimens studied despite the distance of more than 1000 km between the specimens [82]. Venom contains positive inotropic substances that can improve muscle strength by acting directly on the skeletal muscles [83,84]. Fifteen distinct NaTx (To1 to To15) were identified in *Tityus obscurus* venom [72,83]. To1, a β-NaTx, first described as Tc49b in 2001 [85], is the most harmful toxin to humans in the *Tityus obscurus* venom. It enhances the opening probability at more negative potentials of human Na_V_ 1.3 and Nav1.6 [86]. *Tityus metuendus* venom is present in over 200 distinct molecular mass components, including seven α- and β-NaTx interacting with Nav 1.1, Nav 1.2, Nav 1.3, Nav 1.5, and Nav 1.6 (Figure 3B). The venom’s peptides are quite similar to other peptides described in other *Tityus* species, especially the individualized 43, 7, 11, 4, 4, 2, and 14 components corresponding to metalloproteinases, Ktxs, hypothetical proteins, hyaluronidases, endothelin and angiotensin-converting enzymes, allergens, and other peptides (enzymes, proteins, and peptides), respectively [87].

*Buthus occitanus* venom seems to be the most studied venom. However, the modification of the taxonomy in 2017 limits its presence to Europe (France and Spain) [8]. Therefore, only one venom study developed a venom composure to *Buthus occitanus* collected in France [56]. Its venom compounds were mainly between 2500 and 8000 Da, as previously observed in toxic fractions for other Buthidae venom. α-toxins, masses around 2000 Da, over 3000 Da, and over 4000 Da constitute about 10%, 19%, 57 %, and 14% of the venom, respectively (Figure 3C). The venom was mainly composed of α-NaTx (antimammals and anti-insects) but also had a small amount of β-NaTx. The venom reacts with anti-AaH II, anti-Bot I, and anti-AaH IT serum. According to Ktx, an α-KTx3, which is active against Kv1.x, was detected, and the venom reacted with the anti-BmTX3 serum [56]. In mice, the LD50 was 6 μg per mouse and only fractions, immunoreactive to the anti-Bot I serum, were lethal [56].

Other rare types of venom of the scorpion species present in France were also studied. They were limited to *Isometrus maculatus* [88] and *Liocheles australasiae* [89] venom. For other scorpion species, the venom has not been studied, but for some of them, the venom of scorpions of the same genus have be studied (*Centruroides* [90,91,92,93,94,95,96], *Opisthacanthus* [97,98], *Didymocentrus* [99], *Hormurus* [100,101]). In France, these species or genera do not present serious complications during human envenomation, so their venom will not be further developed in the present review.

## 4. Clinical Presentation of Scorpionic Envenomation

### 4.1. Physiopathological Action

The actions of scorpion venom toxins modify cellular action potentials and act on the cardiopulmonary level. They are responsible for direct and indirect cardiac damage through the release of catecholamines and acetylcholine. This mode of action can result in an adrenergic storm that predominantly induces left-sided heart failure and can cause death [76,79,102,103]. Envenomation may be responsible for a muscarinic syndrome characterized by bronchospasms with coughing and wheezing. The plasma levels of lactate, troponin, myoglobin, and brain natriuretic peptides (BNPs) are elevated in response to envenomation [45,104,105]. An electrocardiogram may reveal an isolated prolongation of the QT interval, signs of hypokalemia, or more rarely, hyperkalemia [106]. Acute lung edema induced by scorpionic envenomation may present predominately on the right. There is no clear pathophysiological mechanism which explains the right-lung predominance [107,108]. These complications are often described for envenomation by the *Buthidae* family on French territory, specifically the *Tityus* genera [9].

The NaTxs, KTxs, and calcines of scorpion venom can be distinguished by their effects on neuromuscular action potentials, which causes motor incoordination, muscle spasms, and paralysis at the neurological level [109]. The paralysis is described as ascending from the puncture site, with complete recovery within hours to days occurring in the opposite direction [14,16]. Ophthalmoplegia may be a neurological warning sign of envenomation severity. Patients present with diplopia due to oculomotor muscle paresis, nystagmus, miosis, or mydriasis. This complication may be the combined effect of neurotoxins on the neuromuscular transmission of extraocular muscles, as well as sympathetic or parasympathetic overactivity causing abnormalities in pupillary function [110]. Strokes have been described in patients envenomated by scorpions that do not exist in France. Cerebral infarction, often cerebellar, is due to the release of vasoactive substances or the production of disseminated intravascular coagulopathy [111,112,113]. Neuromuscular disorders are often described for envenomation by the *Buthidae* family in French territory, specifically the *Centruroides* and *Euscorpiidae* genera [9].

In the digestive and urinary tract, NaTxs, KTxs, and calcines induce the release of acetylcholine. Localized abdominal pain and vomiting in the initial phase of envenoming is due to the presence of serotonin in the venom [65]. However, serotonin is not in the composition of all scorpion venom. Cholinergic action then induces a digestive hypersecretion syndrome that is partly responsible for persistent pain, as well as the acceleration of intestinal transit, pancreatitis, or hepatic cytolysis [44,114]. In addition, adrenergic discharge can lead to muscle hypercontractility, which induces acute urine retention and priapism [57]. These complications are often described in envenomation by *Buthidae* on the French territory and by *Tityus* species [9]. The presence of hyperglycemia is a marker of severity that involves the endocrinology system and is associated with significant catecholaminergic release. This reaction results in elevated glucagon and cortisol levels, reduced insulin levels, and insulin resistance [115]. These complications are often described for envenomation by the *Buthidae* family on French territory, specifically the *Centruroides* and *Tityus* genera [9].

At the stinging point, the venom component, excluding NaTx, KTx, or calcine, induces local pain by disrupting the subcutaneous tissue. The increase in vascular permeability and the antiaggregation effect may cause minimal local hematoma. However, the typical appearance of a scorpion sting is a single entry point similar to a needle stick with no other macroscopic features available to the clinician. These complications are often described for envenomation by all French scorpion [9].

### 4.2. Classification of Scorpionic Envenomation

Scorpionic envenoming can result in an adrenergic syndrome, a cholinergic syndrome, and a central neurological syndrome. Several classifications of symptoms and signs of envenomation have been published [1,57,109], with the most widespread and exhaustive classification reported by Khattabi et al. [57]. The severity of scorpionic envenomation is divided into three classes: local symptoms (class I), minor symptoms (class II), and severe symptoms (class III). The symptoms of each class are fully described in Table 2. Hyperleukocytosis, elevated plasma transaminases, lactates, BNP, reduced alkaline reserve, and kalemia are associated with severe envenomation (class III) and a risk of death [45,104,105,114]. The classification of Khattabi et al. does not take the results of complementary examinations (biological, functional, and radiographic) into consideration. Despite this, it is used for the international classification of scorpion envenomation grading in France.

## 5. Management of Scorpionic Envenomation

### 5.1. General Measures for Scorpion Stings

With the exception of French Guiana and the French West Indies, scorpionic envenoming causes minor symptoms in France. However, there are four specific situations that may be fatal and require immediate medical evaluation due to potential complications. These situations include the scorpionic envenomation of a pregnant woman, children under 12 years of age in French Guiana, the development of muscular disorders following envenomation in Guadeloupe, and envenomation by an imported scorpion identified as deadly or not identified.

Local pain can be managed with the application of a local anesthetic, such as eutectic mixtures of lidocaine and prilocaine in cream or patch form. The effectiveness of this approach is superior to that of paracetamol or external cooling, such as the application of an ice bladder, according to a Tunisian study [116]. In some cases, the pain, described as burning or electric shocks, can be very intense and require the prescription of morphine analgesics. Washing and antisepsis of the wound is recommended, as scorpion stings can also be complicated by a local superinfection [9]. This event is rare and does not require prophylactic antibiotics. Scorpionic envenoming does not induce symptoms after 12 h nor does it result in long-term complications [2]. All symptoms improve within hours or days of the envenomation [102].

Some therapies should not be used in the initial phase, including ACE inhibitors and sartans, which worsen the prognosis of patients [117]. Corticosteroids had a limited effect in critically ill envenomated children [118] and also impaired the pulmonary expression of sodium transporters, increasing the risk of pulmonary edema [119]; therefore, we suggest that it should not be recommended. The pharmacological management of other symptoms, such as agitation, diarrhea, hyperthermia, nausea, and vomiting, has not been evaluated. However, the use of antiemetics, antidiarrheals, and other symptomatic treatments does not seem to present any risk, in our experience. Other therapies have not been evaluated and their use remains controversial, such as venom pumps and heat shock. The scorpion venom delivery device is small and, therefore, venom delivery is likely to occur subcutaneously. Indeed, subcutaneous delivery has been described in other venomous species with small venom delivery devices, such as proteroglyph snakes. In the case of a snakebite, a venom pump can extract only a tiny portion (0.04%) [120], which we believe makes it an unfit therapy for the management of envenomation by scorpions. Thermal shock therapy should be performed immediately after envenomation because the small molecules in venom quickly reach their docking sites [37]. In addition, studies on the composition of scorpion venom have not evaluated the thermal stability of the different compounds [63,64], including the venom of French scorpions of medical interest, such as *Buthus occitanus* and *Tityus obscurus* [56,121]. Therefore, we cannot recommend heat shock therapy for scorpionic envenomation.

### 5.2. Specific Measures of Envenomation

The therapeutic management of scorpionic envenomation has historically been based on antivenoms (the only specific treatment), prazosin, dobutamine, and benzodiazepines. The use of a specific antivenom is controversial [116,122,123,124] as it must be considered very early after envenomation since antibodies cannot act on toxins that are irreversibly bound to receptors [37]. These specific treatments are not used for envenomation events in France caused by native scorpions. However, they must be considered for stings by an exotic scorpion in France, as well as for scorpion stings in the French West Indies and in French Guiana. Two antivenoms neutralize the *Tityus* venom (Soro-antiaracnidico^®^ and Soro-antiscorpionico, Instituto Butantan, São Paulo, Brazil) and four others neutralize the *Centruroides* venom (Suero Antialacran^®^, Brimex Intl Co., nanto hsien, Taiwan Ltd., China; Alacramyn^®^, Instituto Bioclon, Mexico City, Mexico; Anascorp^®^, Laboratorios Silanes, Mexico City, Mexico; and Antiscorp^®^, Premium Serums and Vaccines Pvt. Ltd., Maharashtra, India). However, these antivenoms are not available in France. Except in French Guiana, scorpion venoming in France were not fatal and did not induce a long-term sequel. The use of specific antivenoms is not sufficiently beneficial to make them available throughout the territory. In French Guiana, and only in children under 12 years old, an evaluation of the benefit/risk ratio must be carried out to justify its availability.

Prazosin is an alpha blocker. It is prescribed for hypertension caused by catecholaminergic discharge induced by scorpion venom. Its use has been shown to reduce mortality and hospital stays [109,125,126]. The initial dose is 30 μg/kg in children and 0.5 mg in adults, and the maintenance dose is 30 μg/kg every 3 h in children and 0.5 mg every 3 h in adults until a left ventricular ejection fraction over 60% is achieved [109,125,126]. However, its use is authorized in France only for the treatment of hypertension in patients over 12 years. Since it is not authorized for use during “scorpionic envenoming”, its application will be the sole responsibility of the prescriber. Except for nitroglycerin, other vasodilators are not recommended, as they may result in undesirable effects, including the activation of the orthosympathetic system [109].

Dobutamine is indicated for cardiogenic shock, and the optimal efficacy occurs at a dosage of 5 to 10 μg/kg/min [127,128]. This treatment requires management in a critical care unit, as it is a state of shock with acute pulmonary edema that may require mechanical ventilation and sedation [129].

Benzodiazepines seem to reduce the symptom duration of neuromuscular disorders, including fasciculations and dyskinesias. This is especially applicable to cases of envenomation by scorpions of the genus *Tityus* [15,111]; however, their effectiveness has not been proven.

### 5.3. Specificities of Management of Envenoming by Scorpions Responsible of Severe Envenoming

The suspected scorpion envenomation of children (under 12 years of age) in the French West Indies and French Guiana requires urgent medical evaluation. The evaluation identifies signs of severity, such as hemodynamic disorders (tachycardia, bradycardia, and arterial hypotension), respiratory distress (polypnea, dyspnea, and desaturation), and neurological disorders (convulsion, paralysis, vigilance disorder, and coma). The presence of diffuse abdominal pain with or without nausea and hyperglycemia is associated with severe forms of envenomation by *Buthidae* family scorpions [16,102,104]. Hospital monitoring is necessary for at least four hours if any sign is observed, as well as for the detection of delayed symptoms when envenomation has occurred under four hours ago [102]. The absence of symptoms four hours or more after envenomation is associated with a good prognosis and patients no longer require medical monitoring [10,45,102].

The initial management of a patient with even minor signs of envenoming requires hospital monitoring and additional assessments (Table 3). Essential paraclinical examinations include the measurement of capillary glycemia, which indicates hyperglycemia, and an electrocardiogram for the identification of a cardiac rhythm disorder. The recommended biological exams for class II or III envenomation include blood count, blood ionograms, plasma levels of C-reactive protein, lactates, troponin, BNP, and lipase levels. Hyperleukocytosis, hypokalemia, metabolic acidosis, and hyperlactatemia, as well as elevated plasma levels of BNP, troponin, and lipase are signs of severity that require hospitalization [14,15,44,45,46,102,104]. The presence of cough, dyspnea, or crackles on pulmonary auscultation indicate acute pulmonary edema, and a chest X-ray and transthoracic ultrasound should be included. The presence of at least one of these severe criteria warrants management in a critical care unit, and hospital monitoring should be maintained as long as clinical signs persist. All symptoms generally improve in less than 24 h [10,45,102]. Medical management is specified according to envenomation grade in Table 3.

### 5.4. Specific Care of Pregnant and Breastfeeding Women

Scorpion envenoming in pregnant women is rarely described. During pregnancy, scorpion envenoming can induce contractions with a risk of miscarriage or premature birth. Twelve cases have been reported in France between 2012 and 2020, including two envenoming during the third trimester of pregnancy [9]. These last two cases, an envenoming by *Buthus* and one by *Tityus* were marked by contractions, with premature delivery in the case of envenoming by Tityus in French Guiana [45].

The low number of envenoming events in pregnant women does not allow us to draw conclusions about the absence of danger for her and the fetus. However, some studies conducted in other countries and on other species of scorpion suggest that caution should be exercised. Indeed, in the case of delivery triggered by envenoming, it has been described, in Iran, that stillborn babies have been delivered from 22 to 30 weeks due to amenorrhea [130]. This premature delivery is not due to vascular suffering. Indeed, the analysis of envenoming in Taiwan did not objectify a modification of the uterine or placental vascularization after scorpionic envenoming [131]. At birth, fetal distress may be observed in 6 to 11% of births. In utero growth retardation has also been described with rare cases of infants with a low birth weight [130]. These complications are rare and have only been described in Iran after envenoming by *Hemiscorpius lepturus* [130,131,132,133]. On the maternal side, an exceptional case of eclampsia has been described in Turkey [134]. Scorpionic envenoming during pregnancy or the use of antivenom does not appear to have fetotoxicity (assessed up to the 12th month of life) [130,131].

Given the potential complications, envenomed pregnant women should be referred to a local obstetric emergency department for fetal and maternal evaluation before transfer, if necessary, to a higher-level obstetric emergency department. The efficacy of tocolytic treatments for scorpionic envenoming in pregnant women has not, to our knowledge, been studied.

No data are available for breastfeeding women, either at a national or international level. A study on rats described that *Tityus bahiensis* venom affected the development of pups during breastfeeding by envenomed mothers [135]. The absence of human data does not allow us to conclude the effects of venom on breastfeeding women. However, the authors do not recommend breastfeeding after an envenoming.

## 6. Conclusions

Envenomation by native scorpions on the French territories results in mostly local and minor symptoms and treatment is symptomatically focused. However, special surveillance is necessary for cases of scorpion envenomation occurring in the French West Indies, in children under 12 years in French Guiana, and in pregnant women. Prospective studies will help determine the efficacy of antivenoms in overseas territories, as well as prazosin and benzodiazepines in children in French Guiana. They will also be valuable for determining whether tocolytics are useful for pregnant women threatened by premature delivery. The composition of French scorpion venom is poorly known from both a transcriptomic and proteomic level. The study field of this venom is almost virgin and allows for various new discoveries. This is notably the case with the venom of the *Euscorpiidae* family scorpion, which induces muscular disorders, as the venom composition is still unknown.

## Figures and Tables

**Table 2:** International classification of scorpion envenoming according Khattabi and et al. [57].

Class I: Local Symptoms	Class II: Minor Symptoms		Class III: Severe Symptoms
**Local symptoms**	**General symptoms**	**Pulmonary symptoms**	**Neurological failure**
Bullous eruption	Hyperthermia	Stridor	Glasgow Score ≤ 6 (spontaneous)
Burning sensation	Hypothermia	Wheezing	Paralysis
Ecchymosis	Sweating	**Abdominal and urogenital symptoms**	
Erythema	Thirst	Diarrhea	**Cardiogenic failure**
Hyperesthesia	Pallor	Gastrointestinal hemorrhage	Bradycardia
Itching	**Neurological symptoms**	Hematuria	Cardiovascular collapse
Necrosis	Agitation/Restlessness/Excitement	Abdominal distension	Hypotension
Local paresthesia	Anisocoria	Nausea	Ventricular arrhythmia
Pain	Ataxia	Pancreatitis	
Purpura/Petechia	Confusion	Priapism	**Respiratory failure**
Swelling	Convulsion	Urinary retention	Cyanosis
Tingling	Dystonia	Vomiting	Dyspnea
	Encephalopathy	**ENT symptoms**	Pulmonary edema
	Fasciculation	Salivation	
	Headache	Lacrimation	
	Miosis	Odynophagia	
	Mydriasis	Rhinorrhea	
	Nystagmus	Dry mouth	
	General paresthesia	**Muscular symptoms**	
	Prostration	Arthralgia	
	Ptosis	Local muscular cramps	
	Somnolence/Lethargy/Drowsiness	Myoclonia	
	**Cardiovascular symptoms**		
	Hypertension		
	Tachycardia		

**Table 3:** Management of envenoming by scorpions responsible for severe envenoming (class III).

Clinical Presentation	Management	Monitoring Time/Location
**Asymptomatic Patient**		**4 h/Emergency Department (ED)**
General	Washing and disinfection of the wound	
	Measurement of vital parameters, capillary glycemia	
**Class I envenoming**		8 h/ED or hospitalization unit
General	Same as “asymptomatic patient”	
Local pain	Local analgesia: topical anesthetic and external cooling	
**Class II envenoming or presence of isolated hyperglycemia**		**Until symptoms improve (8 h minimum)/hospitalization unit**
General	Same as “asymptomatic patient” and “class I envenoming” Biology: blood count, blood ionogram (K, Na, Cl), Ca, ASAT, ALAT, lipase, urea, creatinine, CRP, troponin, BNP, lactate Frontal chest X-ray 12-lead electrocardiogram (ECG)	
Neuromuscular disorder	Diazepam IVL - Adult: 10 mg - Child: 0.2 mg/kg, not to exceed 10 mg	
Acute pancreatitis	Fasting 72 h Abdominal and pelvic CT scan injected at D3	
Electrocardiographic abnormality	Continuous monitoring by cardio-tensional scope Repeat transthoracic echography (TTE) to assess left heart function	Same/Critical Care Unit
Acute lung edema	Continuous monitoring by cardio-tensional scope Furosemide IVSE - Adult: IVSE 250 to 1000 mg/24 h - Child: IVSE 0.5 to 1 mg/kg/24 h, not to exceed 20 mg Repeat TTE to assess left heart function	Same/Intensive care
Arterial hypertension	Prazosin per os: initial dose then 30 μg/kg/6 h as long as the ventricular ejection fraction is less than 60% - Adult: loading dose and maintenance dose 0.5 mg every 3 h - Child: loading and maintenance dose 30 μg/kg every 3 h Repeat TTE to assess left heart function	Same/Critical Care Unit
**Class III envenoming**		**Until symptoms improve (8 h minimum)/Intensive care**
General	Same as “asymptomatic patient” and “class I and II envenoming” Biology: arterial blood gas 18-lead ECG Repeat TTE to assess left heart function Continuous monitoring by cardio-tensional scope Discuss oro-tracheal intubation to protect the airway	
Cardiogenic shock	Dobutamine 5–10 μg/kg/min (adult and child) Repeat TTE to assess left heart function	
Paralysis	Monitoring of respiratory and swallowing disorders	

## Data Availability

Not applicable.
